# Developmental delay and failure to thrive in a 7-month-old baby boy with spontaneous transient Graves’ thyrotoxicosis: a case report

**DOI:** 10.1186/s13256-016-1013-5

**Published:** 2016-08-10

**Authors:** Shuichi Yatsuga, Tomoko Saikusa, Takako Sasaki, Kikumi Ushijima, Miyuki Kitamura, Junko Nishioka, Yasutoshi Koga

**Affiliations:** Department of Pediatrics and Child Health, Kurume University School of Medicine, 67 Asahi-Machi, Kurume, Fukuoka 830-0011 Japan

**Keywords:** Developmental delay, Failure to thrive, Spontaneous transient Graves’ thyrotoxicosis

## Abstract

**Background:**

Thyroid dysfunction can induce developmental delay and failure to thrive in infancy. Congenital hypothyroidism is one of the common causes of these symptoms in infancy. By contrast, hyperthyroidism is a rare cause of these symptoms in infancy.

**Case presentation:**

A 7-month-old Japanese baby boy was examined for developmental delay and failure to thrive. Blood tests were performed, which showed low levels of thyroid-stimulating hormone (<0.01 μU/mL) and high levels of free thyroxine (2.14 pg/mL). He was referred to our hospital at 8 months of age. His height was 64 cm (–2.7 standard deviation) and his weight was 6085 g (–2.5 standard deviation). No goiter was detected on examination. His thyrotropin receptor antibody was slightly high (3.9 IU/L), whereas thyroid stimulating antibody, anti-thyroglobulin antibody, and thyroid peroxidase antibody were within normal range. These blood findings indicated hyperthyroidism, most likely Graves’ disease. His free thyroxine level decreased in the first month after our examination. No increased vascularity of his thyroid gland was noted. The technetium uptake of his thyroid gland in scintigraphy was relatively increased compared to the intake of his salivary gland. We elected to observe rather than treat with anti-thyroid medications.

**Conclusion:**

We have to rule out spontaneous transient Graves’ thyrotoxicosis when babies have symptoms of developmental delay and fail to thrive.

## Background

Thyroid dysfunction can induce developmental delay and failure to thrive in infancy. Congenital hypothyroidism (CH) is one of the common causes of these symptoms in infancy. By contrast, hyperthyroidism is a rare cause of these symptoms in infancy.

Here we report a case of developmental delay and failure to thrive secondary to spontaneous Graves’ thyrotoxicosis in a 7-month-old baby boy.

## Case presentation

A 7-month-old Japanese baby boy was examined for developmental delay and failure to thrive by a pediatric neurologist. Blood tests were performed and showed that he had a low thyroid-stimulating hormone (TSH) level of <0.01 μU/mL, for which the reference range (rr) is 0.62 to 8.05 μU/mL, and his free thyroxine (FT_4_) level was 2.14 pg/mL (rr 0.48 to 2.34 pg/mL). The pediatric neurologist diagnosed hyperthyroidism and the baby boy was referred to our hospital at 8 months of age.

He was born at 41 weeks of gestation and his weight at birth was 3344 g. His parents were not consanguineous. No familial history of thyroid disease was detected. His height growth and weight gain were poor from 3 months of age. He had hyperthyroid symptoms, such as diarrhea and excessive sweating. His height was 64 cm which was –2.7 standard deviation (SD); his weight was 6085 g (–2.5 SD). His heart rate was 140 beats per minute (bpm; rr 60 to 150 bpm) during sleeping. No goiter was detected on examination. Other general and systemic examinations were unremarkable. Blood tests were performed. His TSH level was <0.05 μU/mL, FT_4_ level was 1.60 pg/mL, and free triiodothyronine (FT_3_) level was 5.2 pg/mL (rr 0.88 to 1.56 pg/mL), total cholesterol level was 112 mg/dL (rr 128 to 219 mg/dL), thyroglobulin level was 73.6 ng/mL (rr 0.0 to 32.7 ng/mL), thyrotropin receptor antibody (TRAb) 3.9 IU/L (rr <1 IU/L), thyroid stimulating antibody (TSAb) 123 % (rr <180 %), anti-thyroglobulin antibody (anti-TgAb) 5.8 IU/mL (rr <9 IU/mL), and thyroid peroxidase antibody (TPOAb) was <0.1 IU/mL (rr <5 IU/mL). These blood findings indicated hyperthyroidism, most likely Graves’ disease (GD). His complete blood count, electrolyte levels, and blood chemistry were within normal range. His FT_4_ level decreased within the first month of our initial examination. No increased vascularity of his thyroid gland was noted. The technetium uptake of his thyroid gland in scintigraphy was relatively increased compared to the intake of his salivary gland. We elected to observe rather than treat with anti-thyroid medications.

One month after our initial examination, his TSH level was 0.274 μU/mL, FT_4_ level was 1.15 pg/mL, and FT_3_ level was 3.8 pg/mL, showing a normal range of thyroid function. We diagnosed this case as spontaneous transient Graves’ thyrotoxicosis. At 3 years of age, the result of a thyroid function test was normal, but a slight developmental delay and failure to thrive were noted: height 85.1 cm (–2.5 SD), weight 10.0 kg (–2.4 SD).

## Discussion

GD is rare in children, with an annual incidence of 0.8 in 100,000, and it is six times more common in girls, thus, GD in boys is very rare [1].

CH is one of the common causes of infant developmental delay and failure to thrive. However, in our case, thyroid function tests revealed hyperthyroidism. Our case did not require methimazole. His C-reactive protein was <0.04 mg/dL, and erythrocyte sedimentation rate was 6.0 mm at 1 hour, his thyroid was not tender or swollen, and TRAb was positive, indicating that we could rule out thyroiditis. His TRAb was at a slightly high level; therefore, he was diagnosed as having GD. His disease course was self-limited and resolved spontaneously. He was diagnosed as having very weak GD or spontaneous transient thyrotoxicosis.

Spontaneous transient thyrotoxicosis with pregnancy occurs in 2 to 3 % of all pregnant women [2]. Two adult cases of spontaneous transient Graves’ thyrotoxicosis without pregnancy were reported [3]. There are no reported cases of spontaneous transient Graves’ thyrotoxicosis in children.

## Conclusions

We reported a case of developmental delay and failure to thrive in a baby boy caused by spontaneous transient Graves’ thyrotoxicosis. This was treated with observation and resolved spontaneously.

## Abbreviations

anti-TgAb, anti-thyroglobulin antibody; bpm, beats per minute; CH, congenital hypothyroidism; FT_3_, free triiodothyronine; FT_4_, free thyroxine; GD, Graves’ disease; rr, reference range; SD, standard deviation; TPOAb, thyroid peroxidase antibody; TRAb, thyrotropin receptor antibody; TSAb, thyroid stimulating antibody; TSH, thyroid-stimulating hormone
